# Eosinophilic Myocarditis due to Toxocariasis: Not a Rare Cause

**DOI:** 10.1155/2016/2586292

**Published:** 2016-03-31

**Authors:** Shunichi Shibazaki, Shunsuke Eguchi, Takashi Endo, Tadamasa Wakabayashi, Makoto Araki, Yoshiaki Gu, Taku Imai, Kouji Asano, Norihide Taniuchi

**Affiliations:** ^1^Department of Internal Medicine, Suwa Central Hospital, 4300 Tamagawa, Chino, Nagano 391-8503, Japan; ^2^Department of Cardiology, Nagoya University Hospital, 65 Tsurumai, Showa, Nagoya, Aichi 466-8560, Japan; ^3^Department of Internal Medicine, Ishinomaki City Hospital, 25-1 Minamisakai-Shinkozutsumi, Ishinomaki, Miyagi 986-0031, Japan; ^4^Department of Cardiology, Suwa Central Hospital, 4300 Tamagawa, Chino, Nagano 391-8503, Japan; ^5^Department of Infectious Disease, Tohoku University Hospital, 1-1 Seiryou, Aoba, Sendai, Miyagi 980-0872, Japan; ^6^Department of Pathology, Suwa Central Hospital, 4300 Tamagawa, Chino, Nagano 391-8503, Japan

## Abstract

Myocarditis is a clinically important disease because of the high mortality. From the perspective of treatment strategy, eosinophilic myocarditis should be distinguished from other types of myocarditis. Toxocariasis, caused by* Toxocara canis* or* Toxocara cati*, is known as a cause of eosinophilic myocarditis but is considered rare. As it is an unpopular disease, eosinophilic myocarditis due to toxocariasis may be underdiagnosed. We experienced two cases of eosinophilic myocarditis due to toxocariasis from different geographical areas in quick succession between 2013 and 2014. Case 1 is 32-year-old man. Case 2 is 66-year-old woman. In both cases, diagnosis was done by endomyocardial biopsy and IgG-ELISA against* Toxocara* excretory-secretory antigen. Only a corticosteroid was used in Case  1, whereas a corticosteroid and albendazole were used in Case  2 as induction therapy. Both patients recovered. Albendazole was also used in Case  1 to prevent recurrence after induction therapy. Eosinophilic myocarditis by toxocariasis may in actuality not be a rare disease, and corticosteroid is an effective drug as induction therapy even before use of albendazole.

## 1. Introduction

Myocarditis is a serious clinical situation that is considered rare as it is found in only 0.1% of pathological autopsies [1]. However, it has a high mortality rate (38–54%) [2]. It is important to distinguish subtypes of this diagnosis in order to better understand the role of corticosteroids or other therapies that may be effective in treating myocarditis.

Eosinophilic myocarditis comprises 6% of all types of myocarditis [3], and most cases of eosinophilic myocarditis are described as idiopathic. The frequency of other causes of eosinophilic myocarditis is not well understood, and there are only 14 reports of eosinophilic myocarditis due to toxocariasis, caused by* Toxocara canis* (*T. canis*) or* Toxocara cati* (*T. cati*) [4].

We experienced two cases of eosinophilic myocarditis due to toxocariasis from different geographical areas between 2013 and 2014, which suggests that the actual frequency of eosinophilic myocarditis due to toxocariasis has been underestimated. The reports presented here indicate that it may be helpful to use corticosteroids as induction therapy for eosinophilic myocarditis due to toxocariasis.

## 2. Case Report

*Case  1*. The first case was a 32-year-old Japanese man who lived in Tianjin, China, for business. His job was in sales for motor parts. Upon returning to Japan, he went to a hospital for chest discomfort and dyspnea following exercise in December 2013. He had ulcerative colitis and the activity was controlled without medication. His last recurrence of ulcerative colitis was unknown. He had no history of allergy and no pets.

The initial examination showed the following findings: blood pressure of 122/68 mmHg and heart rate of 83 beats/min without cardiac gallop rhythm.

**Figure 1:**
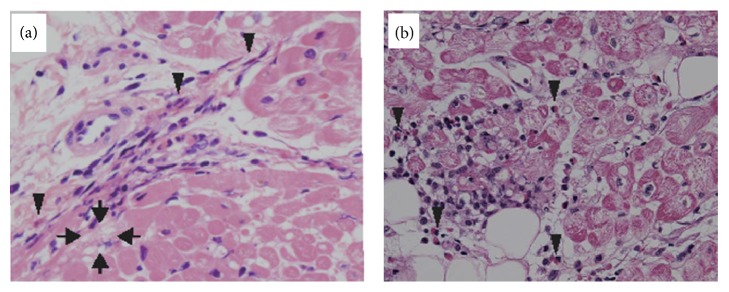
Endomyocardial biopsy of Case  1 (a) and Case  2 (b) (H-E staining; original magnification ×400). Focal and mild infiltration of eosinophil with degranulation (arrowhead) was seen in the interstitium of the myocardium. Some myocytes appeared degenerative (arrow).

**Figure 2:**
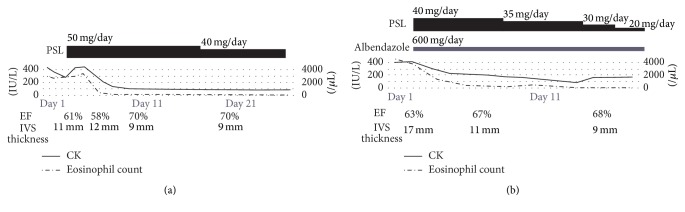
Clinical courses of the cases. (a) Case  1. (b) Case  2. In both cases, after starting prednisolone, CK and eosinophil count immediately came into the normal range, and ejection fraction and wall thickness also recovered. CK, creatinine phosphokinase; EF, ejection fraction; IVS, interventricular septum thickness; PSL, prednisolone.

Laboratory examination on admission revealed an increase in the total white blood cell count (12,540/*μ*L), especially due to an increase in the eosinophil count (2,880/*μ*L) and elevated enzymes: creatinine phosphokinase (CK) of 303 IU/L and Troponin-I of 1.863 ng/mL. Electrocardiogram (ECG) showed an abnormal Q wave in III and aVR leads and a negative T wave in II, III, aVF, V5, and V6 leads. Chest X-ray (CXR) showed cardiac dilation. Transthoracic echocardiography (TTE) showed thickening of the left ventricular (LV) wall; in particular, intraventricular wall thickness was 11-12 mm, with global hypokinesis. There was no significant valvular dysfunction and pericardial effusion. Contrast enhanced computed tomography (CT) of the chest showed a focal deficit in the myocardium but no deficit in the pulmonary artery. Coronary angiography (CAG) was normal. Endomyocardial biopsy showed eosinophil invasion into the myocardium (Figure 1(a)). Although not intense, the degranulated eosinophil infiltration with few other inflammatory cells indicated that there was significant active eosinophilic inflammation. As a whole, we diagnosed this as eosinophilic myocarditis. A high dose of corticosteroid, prednisolone (PSL) 1 mg/kg/day, was used orally as induction therapy. After using PSL, the eosinophilia disappeared within several days and his cardiac symptoms, CK, LV wall thickening, and ejection fraction recovered in parallel (Figure 2(a)). During this period, ECG and TTE changed variously, and cardiac dilation disappeared in CXR. Hemodynamics were maintained consistently. Later, we suspected parasite infection because of unknown cause of eosinophilic myocarditis. We examined a commercial multiple-dot ELISA kit (SRL, Tokyo, Japan) as a screening test; anti-IgG to* T. canis* was detected in his serum. For further study to confirm the diagnosis, we performed IgG-ELISA to* Toxocara* excretory-secretory antigen (TES-IgG-ELISA) at the Department of Parasitology, Miyazaki Medical University (Miyazaki, Japan). The TES-IgG-ELISA consisted of the following: wells of microtiter plates were coated with 10 *μ*g/mL of* T. canis* larval excretory-secretory antigen and intubated with diluted samples (1 : 900–1 : 2,700). Binding of antibodies to* T. canis* antigen was detected with horseradish peroxidase-conjugated anti-human IgG and optical densities were read with a microplate reader. We detected TES-IgG-ELISA in his serum. We did not examine* T. canis* larval excretory-secretory antigen with Western blotting. Taken together, the patient's background, high prevalence of* Toxocara* spp. in China, clinical data, especially eosinophilia, and high presence of* T. canis* antibody led to our diagnosis of myocarditis by toxocariasis. Although he was almost free of cardiac symptoms, we added an anthelmintic, albendazole (600 mg/day orally), to his treatment regimen from day 44. For two months after induction therapy, physical examination, laboratory tests, and ECG showed normal findings. He subsequently returned to China. Subsequent course was unknown.

*Case  2*. This second case was a 66-year-old Japanese woman living in Nagoya, Japan, who had never been abroad. She had a fever of unknown origin, chronic diarrhea, elevated biliary enzyme, and eosinophilia for a month in June 2014. We suspected various differential diagnosis such as the following because of fever of unknown origin with eosinophilia; allergy, drug side effect, HIV infection, chronic eosinophilic leukemia, eosinophilic granulomatosis with polyangiitis, and so forth. She did not have allergic disorders and other new drugs. We also ruled out other diseases serologically, including HIV infection and chronic eosinophilic leukemia and eosinophilic granulomatosis with polyangiitis. We suspected parasite infection because she was a cook and often used vegetables from an open-field culture. We examined a commercial multiple-dot ELISA kit (SRL, Tokyo, Japan) as a screening test. Because anti-IgG to* T. canis* was positive, we performed TES-IgG-ELISA at the Department of Parasitology, Miyazaki Medical University (Miyazaki, Japan). TES-IgG-ELISA was also positive in her serum. Taken together, our diagnosis was visceral larva migrans due to toxocariasis. We planned to use an anthelmintic, albendazole, in a few days. Six weeks later from disease onset, before using albendazole, in August 2014, she was admitted to the hospital for chest discomfort and presyncope. She had chronic kidney disease (CKD) stage 5, persistent atrial fibrillation, and chronic heart failure.

The initial examination showed blood pressure of 100/75 mmHg, and she had a third heart sound. Laboratory data on admission revealed an increase in the total white blood cell count (9,700/*μ*L), along with a high eosinophil count (4,510/*μ*L). She also had elevated enzymes: CK of 433 IU/L and Troponin-T of 6.31 ng/mL. ECG showed a complete right bundle branch block that had not been seen before. CXR showed cardiac dilation. TTE showed that the LV wall was hyperechoic and thickening; in particular, intraventricular wall thickness was 17 mm. CAG was not done because of the CKD 5 stage; however, endomyocardial biopsy was done. Endomyocardial biopsy showed eosinophil invasion to the myocardium; some eosinophils were degranulated (Figure 1(b)). Our diagnosis was eosinophilic myocarditis due to toxocariasis. Both prednisolone (PSL, 1 mg/kg/day orally) and albendazole (600 mg/day orally) were used as induction therapy. Eosinophilia disappeared within several days and her cardiac symptoms, CK, and LV wall thickening recovered in parallel (Figure 2(b)). After the induction therapy, ECG and TTE changed variously and cardiac dilation improved slightly; however, it remained in CXR. Hemodynamics were stable consistently. We used albendazole for 4 weeks and decreased PSL gradually with 5 mg PSL as a maintenance dose. She did not have recurrence after 4 months from onset.

## 3. Discussion

These two cases of eosinophilic myocarditis were caused by toxocariasis. This report shows two clinically important observations. First, toxocariasis is often overlooked as a cause of eosinophilic myocarditis; therefore, the frequency of reports on toxocariasis causing this disease may be inaccurate. Second, corticosteroids may be a key drug for treating eosinophilic myocarditis even when caused by toxocariasis.

Myocarditis is serious because it may cause acute heart failure, cardiogenic shock, and arrhythmia. Although it is found at only 0.1% in pathological autopsies [1], the actual mortality rate is quite high (38–54%) [2]. Myocarditis is divided into four subtypes according to pathological findings: lymphocytic, giant cell, eosinophilic, and granulomatous. Eosinophilic myocarditis comprises 6% of all types of myocarditis [3]. In addition, eosinophilic myocarditis may often be caused by another background disease which is idiopathic or vasculitis, neoplasms, parasites, and drugs [5]. It is important to clinically distinguish eosinophilic myocarditis from other types of myocarditis to offer the most effective therapy.

Toxocariasis may be overseen as a cause of eosinophilic myocarditis. One report shows that 30% of eosinophilic myocarditis is idiopathic. On the other hand, visceral larva migrans caused by all types of parasites represents only 15% of eosinophilic myocarditis [6]. However, it is possible that toxocariasis is misdiagnosed and the cause of eosinophilic myocarditis is diagnosed as idiopathic. One reason for misdiagnosis is that* Toxocara* spp. infection itself in humans is almost always asymptomatic.* Toxocara* spp. affect dogs (*T. canis*) and cats (*T. cati*), which excrete invective ova in their feces. Human can be infected by ingesting embryonated* Toxocara* eggs from vegetables contaminated by the infected dog or cat feces or by eating undercooked meat from paratenic hosts. The eggs hatch and larvae penetrate the intestinal wall, reaching the liver through the portal vein.* Toxocara* spp. can also reach other organs like the heart through systemic circulation. The frequency of* T. canis* infection in adult dogs is 4.3% in Japan [7]. However,* Toxocara* spp. infection in adult humans is increasing with the recent increase in pets. In fact, antibodies against* Toxocara* spp. are confirmed in 1–6% of healthy Japanese people [8] and 13% of healthy Chinese people [9].* Toxocara* spp. infection sometimes occurs, but many physicians may not be familiar with it; therefore, it is a neglected parasitic infection [10]. Incidentally, we cannot distinguish between* T. canis* and* T. cati* by TES-IgG-ELISA or Western blot due to antibody cross-reactivity [11, 12]. In addition, toxocariasis may lead to eosinophilic myocarditis to a greater extent than we imagine. In a laboratory experiment of mice, 10–15% of mice infected by* T. canis* developed eosinophilic myocarditis [10]. We reported on two cases that occurred within a two-year period in different geographical areas. This suggests that the current reported frequency of eosinophilic myocarditis caused by* T. canis* infection may be lower than actual situation.

Corticosteroids rather than albendazole may be an effective treatment for eosinophilic myocarditis caused by toxocariasis in acute-phase treatment. In Case  1, use of only the corticosteroid without albendazole achieved remission. Previous experiments on animals also show that corticosteroids, rather than albendazole, seem to be effective for the following reason. In mice diagnosed with eosinophilic myocarditis due to* T. canis*, the inhibition of eosinophil invasion to myocardium prevented myocardial damage or myocardial fibrosis without clearing* T. canis* [13]. This report shows that eosinophilia itself rather than* Toxocara* spp. invasion to myocardium is essential in eosinophilic myocarditis, and, in this sense, eosinophil suppression is significant for acute-phase treatment. Possibly, albendazole may contribute as chronic-phase treatment to prevent eosinophilia or eosinophilic myocarditis.

More case reports are needed to accurately capture the frequency of eosinophilic myocarditis and its appropriate treatment for both acute-phase treatment and chronic-phase treatment to prevent relapse. Patients should be tested for* Toxocara* spp. infection properly in order to establish the connection between this infection and eosinophilic myocarditis.

## 4. Conclusions

The two cases presented here suggest that toxocariasis is often overlooked as a cause of eosinophilic myocarditis. Therefore, clinicians should check TES-IgG-ELISA when eosinophilic myocarditis is suspected. In addition, a corticosteroid may be a key drug for treating eosinophilic myocarditis due to toxocariasis in the acute phase even before use of albendazole.
